# Experimental validation of contrast-enhanced SSFP cine CMR for quantification of myocardium at risk in acute myocardial infarction

**DOI:** 10.1186/s12968-017-0325-y

**Published:** 2017-01-30

**Authors:** David Nordlund, Mikael Kanski, Robert Jablonowski, Sasha Koul, David Erlinge, Marcus Carlsson, Henrik Engblom, Anthony H. Aletras, Håkan Arheden

**Affiliations:** 1 Department of Clinical Physiology, Clinical Sciences, Lund University and Lund University Hospital, Lund, Sweden; 2 Department of Cardiology, Clinical Sciences, Lund University and Lund University Hospital, Lund, Sweden; 3 Laboratory of Computing and Medical Informatics, School of Medicine, Aristotle University of Thessaloniki, Thessaloniki, Greece

**Keywords:** Myocardium at risk, CE-SSFP, AAR, Area at risk

## Abstract

**Background:**

Accurate assessment of myocardium at risk (MaR) after acute myocardial infarction (AMI) is necessary when assessing myocardial salvage. Contrast-enhanced steady-state free precession (CE-SSFP) is a recently developed cardiovascular magnetic resonance (CMR) method for assessment of MaR up to 1 week after AMI. Our aim was to validate CE-SSFP for determination of MaR in an experimental porcine model using myocardial perfusion single-photon emission computed tomography (MPS) as a reference standard and to test the stability of MaR-quantification over time after injecting gadolinium-based contrast.

**Methods:**

Eleven pigs were subjected to either 35 or 40 min occlusion of the left anterior descending artery followed by six hours of reperfusion. A technetium-based perfusion tracer was administered intravenously ten minutes before reperfusion. In-vivo and ex-vivo CE-SSFP CMR was performed followed by ex-vivo MPS imaging. MaR was expressed as % of left ventricular mass (LVM).

**Results:**

There was good agreement between MaR by ex-vivo CMR and MaR by MPS (bias: 1 ± 3% LVM, *r* ^2^ = 0.92, *p* < 0.001), between ex-vivo and in-vivo CMR (bias 0 ± 2% LVM, *r* ^2^ = 0.94, *p* < 0.001) and between in-vivo CMR and MPS (bias -2 ± 3% LVM, *r* ^2^ = 0.87, *p* < 0.001. No change in MaR was seen over the first 30 min after contrast injection (*p* = 0.95).

**Conclusions:**

Contrast-enhanced SSFP cine CMR can be used to measure MaR, both in vivo and ex vivo, in a porcine model with good accuracy and precision over the first 30 min after contrast injection. This offers the option to use the less complex ex-vivo imaging when determining myocardial salvage in experimental studies.

## Background

Treatment options for acute myocardial infarction (AMI) have steadily increased over the recent decades. Experimental models have been instrumental to this since they offer a controlled environment where treatment effects can be developed and assessed prior to patient trials. An important endpoint for such models is myocardial salvage and myocardial salvage index (MSI) [1], used to quantify treatment efficacy. Myocardial salvage is defined as myocardium at risk (MaR) minus infarct size and MSI is defined as myocardial salvage divided by MaR.

Cardiovascular magnetic resonance (CMR) is considered the reference standard for determining infarct size in vivo using late gadolinium enhancement (LGE) imaging typically performed 15–20 min after contrast administration [2, 3]. Furthermore, CMR has been shown to enable assessment of MaR using T2-weighted (T2w) imaging which has been validated both in an experimental AMI model [4] and in patients up to 1 week after AMI [5]. However, T2w imaging performs inconsistently depending on vendor [6] and on the particular implementation of the T2w pulse sequence. Also, T2w imaging has been shown to be prone to artefacts [7, 8]. Another CMR method for MaR quantification is contrast-enhanced steady state free precession (CE-SSFP), which is based on standard SSFP cine images acquired after contrast injection(9). Since SSFP cine imaging performs consistently for all vendors and since images are included in the majority of standard CMR protocols for assessment of cardiac function, CE-SSFP can be implemented across centers and does not add to the total scanning time [6]. CE-SSFP has been validated in patients with myocardial perfusion single-photon emission computed tomography (MPS) [9], compared head-to-head with T2w imaging [10] and used in two multinational cardio-protection trials, CHILL-MI and MITOCARE [11, 12]. However, to date CE-SSFP has not been validated experimentally.

The specific aims for this study were: A. To determine whether CE-SSFP could be used for determining MaR in an ex-vivo setting, which would be advantageous for designing cardioprotective studies where performing complex in-vivo CMR is not possible. B. To test if the findings in patients with respect to quantification of MaR with CE-SSFP would also hold in an in-vivo animal model and C. To determine whether the timing of imaging after contrast agent administration affects the quantification of MaR in vivo.

## Methods

### Experimental model

The study was approved by the local Ethics Committee for animal experiments. Pigs (weighing 40–50 kg) were pre-medicated with ketamine 15 mg/kg (Ketaminol, Intervet, Danderyd, Sweden) and midazolam 0.5 mg/kg intramuscularly (Dormicum, Roche AB, Stockholm, Sweden) after overnight fasting with free access to water. Anesthesia was induced with propofol 20 mg/ml (Propofol Sandoz A S, Copenhagen, Denmark) and the animals were intubated using cuffed endotracheal tubes. Anesthesia was maintained with inhalation of sevoflurane gas (Sevorane, Baxter Medical AB, Kista, Sweden) using a disposable administration system (AnaConDa, Sedana Medical AB, Uppsala, Sweden) and titrating to desired effect. Mechanical ventilation was established using a 900C ventilator (Siemens AB, Upplands Väsby, Sweden) in volume controlled mode regulated to a pCO_2_ of 5.0–6.0 kPa. Monitoring included arterial blood pressure, heart rate, ECG, pulse-oximetry and temperature. Arterial blood gases were drawn and analyzed directly after establishing arterial access, before occlusion, after reperfusion, and to follow up any unexpected deviation of the blood gas results or change in the clinical condition of the animal. Venous and arterial femoral access, jugular access and carotid access were established using 6–8Fr introducer sheaths. After establishing all accesses, 20,000 IU of heparin (LEO Pharma AB, Malmö Sweden) was administered intravenously and 5% glucose (Baxter Medical AB, Kista, Sweden) was slowly infused. During the experiments amiodarone (Sanofi AB, Stockholm, Sweden), fentanyl (50 mikrog/ml, B. Braun Medical AB, Danderyd, Sweden) and sodium chloride (0.9% Baxter Medical AB, Kista, Sweden) was titrated to desired effect.

### Experimental protocol

Pigs were subjected to either 35 or 40-min of left anterior descending (LAD)-occlusion using a balloon-tipped catheter, followed by six hours of reperfusion. The balloon occluder was placed either after the first or the second diagonal branch of the LAD to obtain a wide range of MaR. An angiogram was acquired after inflation of the balloon and before deflation in order to confirm occlusion of the coronary vessel and correct balloon placement. Isotope (1000 MBq ^99m^Tc tetrofosmine) for MPS imaging was administered ten minutes prior to reperfusion. After deflation of the balloon a subsequent angiogram was performed to verify restoration of blood flow. After reperfusion, animals were transported to the MR department.

### CMR

#### In vivo

Imaging was performed on a 1.5 T MR scanner (Philips Achieva, Best, Netherlands) using a 32-channel cardiac coil. During in-vivo imaging 0.2 mmol/kg gadolinium (Gd) based contrast agent (Gd-DOTA) (Dotarem, Guerbet, Roissy, France) was administrated intravenously 15 min before LGE-CMR. Using a retrospectively gated SSFP single slice sequence, a midventricular short-axis slice of the left ventricle (LV) was repeatedly acquired during the first ten minutes after contrast injection. Then, a short-axis stack covering the entire LV was acquired, using the same sequence parameters as for the single slice (typical parameters: TE 1.40 ms, TR 2.8 ms, flip angle 60°, 25 phases, slice thickness 8 mm, no slice gap, acquisition matrix 230 x 230, reconstructed matrix 240 x 240, FOV 320 x 320 mm, pixel bandwidth 860 Hz/pixel). LGE images were acquired 15–20 min after contrast injection using an inversion recovery gradient echo sequence (typical parameters: TE 3.0, TR 6.1 ms, TI 320 ms, slice thickness 8 mm, no slice gap, acquisition matrix 200 x 158, reconstructed matrix 510 x 510, FOV 320 x 320 mm, pixel bandwidth 260 Hz/pixel).

After CE-SSFP and LGE imaging an additional single slice of SSFP in the same position as above was acquired 20–30 min after contrast injection. Both CE-SSFP and LGE slices were obtained during end-expiratory breath hold. Long axis 2-, 3 and 4-chamber views using both CE-SSFP and LGE were also acquired. Fifteen minutes before euthanization an additional 0.2 mmol/kg Gd-DOTA was administrated. Pigs were euthanized with a rapid infusion of saturated potassium chloride solution and the hearts were explanted and suspended in plastic containers with deuterated water-filled balloons in the ventricles for ex-vivo imaging.

#### Ex vivo

ex-vivo CMR was performed on the same scanner as above, using a simulated ECG with heart rate 60 beats per minute for triggering. A full coverage LV short-axis stack was acquired using the same settings as for the in-vivo sequence above (typical parameters: TE 1.40 ms, TR 2.8 ms, flip angle 60°, 25 phases, slice thickness 8 mm, no slice gap, acquisition matrix 60 x 50, reconstructed matrix 80 x 80, FOV 100 × 100 mm, pixel bandwidth 1400 Hz/pixel). A high resolution T1-weighted short axis stack was acquired for detailed infarct visualization (TE 3.4 ms, TR 20 ms, 0.5 mm isotropic voxels, no slice gap, acquisition matrix 200 × 200, reconstructed matrix 220 × 220, FOV 100 × 100 mm, pixel bandwidth 440 Hz/pixel).

### MPS

ex-vivo MPS was performed approximately 8–10 h after intravenous injection of a 1000 MBq dose of ^99m^Tc-tetrofosmin using a dual head camera (Philips SKYlight, Best, the Netherlands) and a vertex high resolution collimator (ADAC Vertex, Milpitas, CA, USA) at 32 projections (40 s per projection) with a 64 × 64 matrix yielding a digital resolution of 4.24 mm isotropic voxels. Iterative reconstruction using maximum likelihood expectation maximization (MLEM) was performed with a low resolution Butterworth filter with a cut-off frequency set to 0.6 of Nyquist and order 5.0. No attenuation or scatter correction was applied. Finally, a short-axis image stack was reconstructed using commercially available software (AutoSPECT Plus, Pegasys software version 5.01, Philips, Best, The Netherlands).

### Image analysis

All images were analyzed using the software Segment, version 1.9 R3314 (http://segment.heiberg.se) [13]. MaR from the in-vivo CE-SSFP images was assessed according to a previously described method [9]. In short, LVM was defined by manual delineation of the epicardial and endocardial borders in end-diastolic and end-systolic timeframes. Hyperintense myocardium was then manually delineated and defined as MaR. The same method was used to delineate MaR in ex-vivo CE-SSFP images. The analysis of CE-SSFP images was performed both by a blinded primary observer and an independent, blinded secondary observer. When delineating CE-SSFP images over time after injection of gadolinium all images were sorted randomly both between timepoints and between experiments and observers were blinded to the randomization.

Infarcted myocardium was delineated from the in-vivo short-axis LGE images according to a previously defined method [14]. In short, the endocardial and epicardial borders were manually traced with exclusion of the papillary muscles. The LGE myocardium was defined using a computer algorithm that takes into consideration partial volume effects within the infarcted region [14]. Manual adjustments were made when image artefacts caused misinterpretation by the computer algorithm. Hypointense signal within the area of LGE, microvascular obstruction [15], was included and considered as infarction.

The high resolution T1-weighted ex-vivo images were also delineated according to a previously described and validated method [16]. In short, the endocardial and epicardial borders were traced manually including the papillary muscles. Infarct was defined as >8 standard deviations from a manually defined remote region. Manual adjustments were made if the computer algorithm was clearly wrong due to image artefacts or inclusion of intramyocardial fat as infarct.

MaR and infarct size was expressed as percentage of LVM. Myocardial salvage index (MSI) was calculated as (MaR-infarct size)/MaR = MSI.

Evaluation of MPS images was performed by using anatomical information from the high resolution T1-weighted ex-vivo images and perfusion information from MPS according to a previously described method [17]. In short, both T1-weighted and MPS images were re-sampled to similar resolutions and were spatially matched using purpose-designed software. Delineations of endocardium and epicardium from the T1-weighted images were then used for defining the myocardium in the MPS images. MaR was then automatically defined by calculating and applying a threshold and performing manual adjustments as previously described [17].

### Statistics

Analyses were performed using GraphPad Prism (version 6.00, GraphPad Software, San Diego, USA) or IBM SPSS Statistics (version 23, IBM Corporation, New York, USA). Results for continuous variables are expressed as mean ± standard deviation. Bias according to Bland-Altman was used to compare MaR by MPS, in-vivo CE-SSFP and ex-vivo CE-SSFP. Bias according to Bland Altman was also used to compare MaR by MPS, in-vivo CE-SSFP and ex-vivo CE-SSFP to infarct size by LGE imaging [18], and for inter-observer analysis. A paired *t*-test was used to test ex-vivo CE-SSFP vs MPS and in-vivo CE-SSFP vs ex-vivo CE-SSFP. Pearson correlation coefficient was used for assessment of correlation between ex-vivo CE-SSFP, MPS, in-vivo CE-SSFP, infarct, and for inter-observer analysis. When comparing MaR over time for the single-slice images, the MaR was normalized to MaR of the first acquired slice. Subsequently a repeated measures linear mixed model using time as a nominal variable and a fixed effect was used to test for differences in MaR between different time points after contrast injection. *P* < 0.05 indicated statistical significance.

## Results

### Study population

Twelve pigs were included in the study. In one pig no infarct or MaR could be identified neither with in-vivo or ex-vivo CMR nor with MPS. In two pigs the in-vivo images were considered of non-diagnostic quality due to severe arrhythmias and high heart rate (>120 bpm). MPS images were of diagnostic quality in all pigs.

### MaR

**Fig. 1:**
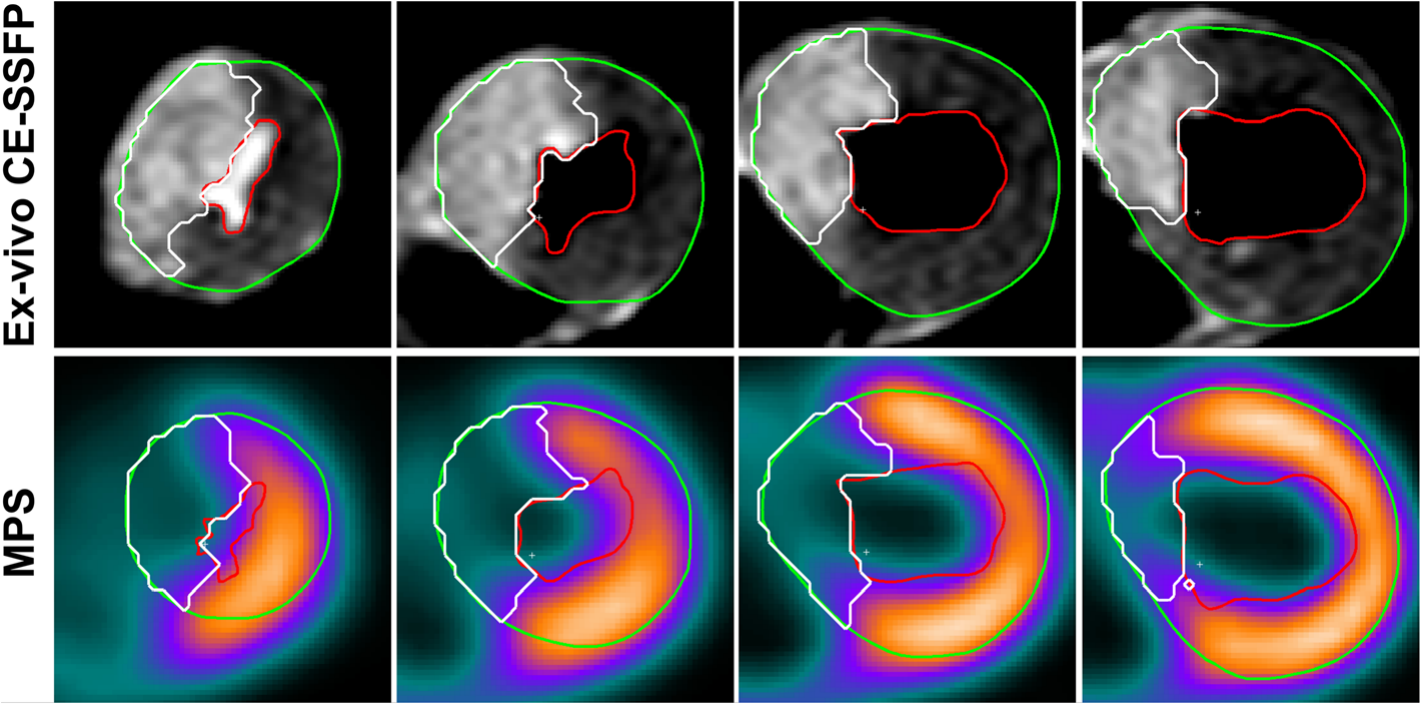
Myocardium at risk (MaR) is shown to have similar extent with ex-vivo CE-SSFP (*top row*) and MPS (*bottom row*) in matched slices from the same heart. The *green line* denotes epicardium, the *red line* endocardium and the white line MaR. MPS = myocardial perfusion single photon emission tomography

**Fig. 2:**
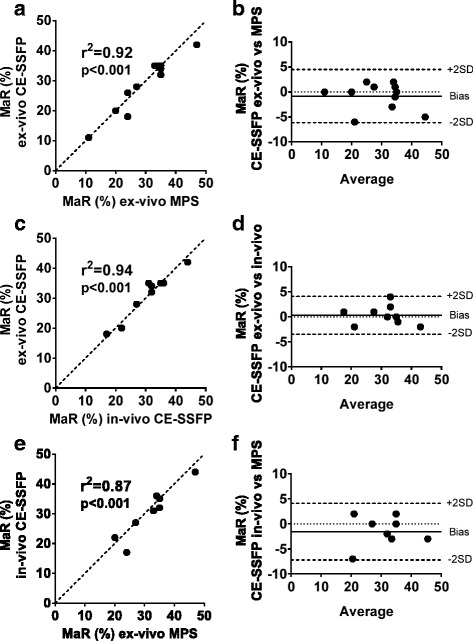
Myocardium at risk (MaR) by MRI and MPS show overall good correlation and agreement. Panel **a** shows a good correlation between MaR by ex-vivo CE-SSFP and MaR by myocardial perfusion SPECT (MPS). The corresponding Bland-Altman plot in panel **b** shows a low bias. Panel **c** shows a good correlation between MaR by ex-vivo CE-SSFP and MaR by in-vivo CE-SSFP, with a low bias (panel **d**). Panel **e** shows a good correlation between MaR by in-vivo CE-SSFP and MaR by MPS. The corresponding Bland-Altman plot in panel **f** shows a low bias. In all cases, MaR is expressed as percent of left ventricular mass. The *dotted lines* in a, c and e represent the line of identity

Images of MaR by ex-vivo CE-SSFP and MPS from one pig of typical quality is shown in Fig. 1. A good correlation with a low bias was found between MaR by ex-vivo CE-SSFP (29 ± 9%) and MPS (30 ± 9%) (*n* = 11, bias: 1 ± 3%, *r* ^2^ = 0.92, *p* < 0.001, Fig. 2). There was also a good correlation with a low bias between MaR by in-vivo CE-SSFP (31 ± 8%) and ex-vivo CE-SSFP (*n* = 9, bias: 0 ± 2%, *r* ^2^ = 0.94, *p* < 0.001, Fig. 2). MaR by in-vivo CE-SSFP vs MPS showed a low bias (-2 ± 3%) and a good correlation (*n* = 9, *r* ^2^ = 0.87, *p* < 0.001, Fig. 2). Inter-observer analysis for the ex-vivo CE-SSFP showed a low bias (*n* = 11, bias: -1.5 ± 3.7%, *r* ^2^ = 0.98). For the in-vivo CE-SSFP bias was also low (*n* = 9, bias: -0.4 ± 4.3%, *r* ^2^ = 0.77).

### Infarct

**Fig. 3:**
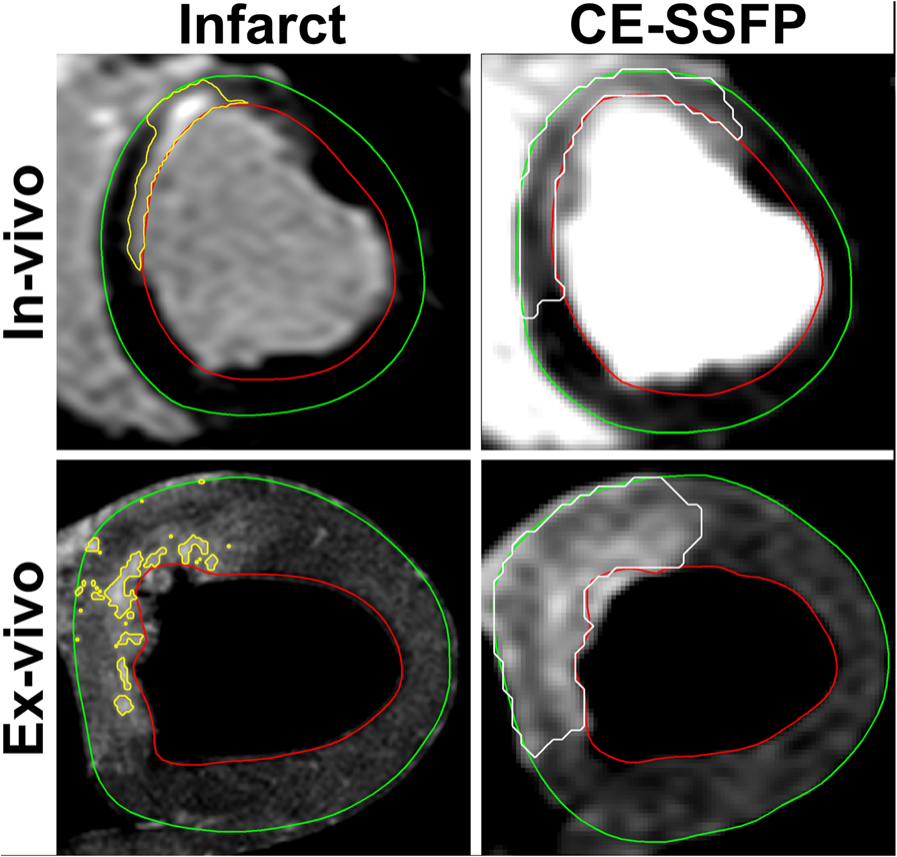
Infarct and myocardium at risk (MaR) in vivo and ex vivo. The upper row shows infarct by late gadolinium enhancement (LGE) and MaR by CE-SSFP in vivo and the lower row shows infarct by high-resolution T1-weighted imaging and MaR by CE-SSFP ex vivo. Note the significantly larger MaR compared to infarction both in vivo and ex vivo

Examples of infarct- and MaR-images in vivo and ex vivo of typical quality are seen in Fig. 3. Infarct size was 13 ± 10% by high-resolution T1-weighted ex-vivo imaging. Infarct size on average was smaller than MaR by MPS, CE-SSFP ex vivo and CE-SSFP in vivo in all pigs (mean difference 17 ± 10%, 15 ± 8% and 16 ± 9% respectively).

### Myocardial salvage Index

Myocardial salvage index was 52 ± 28% by ex-vivo CE-SSFP and high resolution T1-weighted ex-vivo imaging.

### MaR over time

**Fig. 4:**
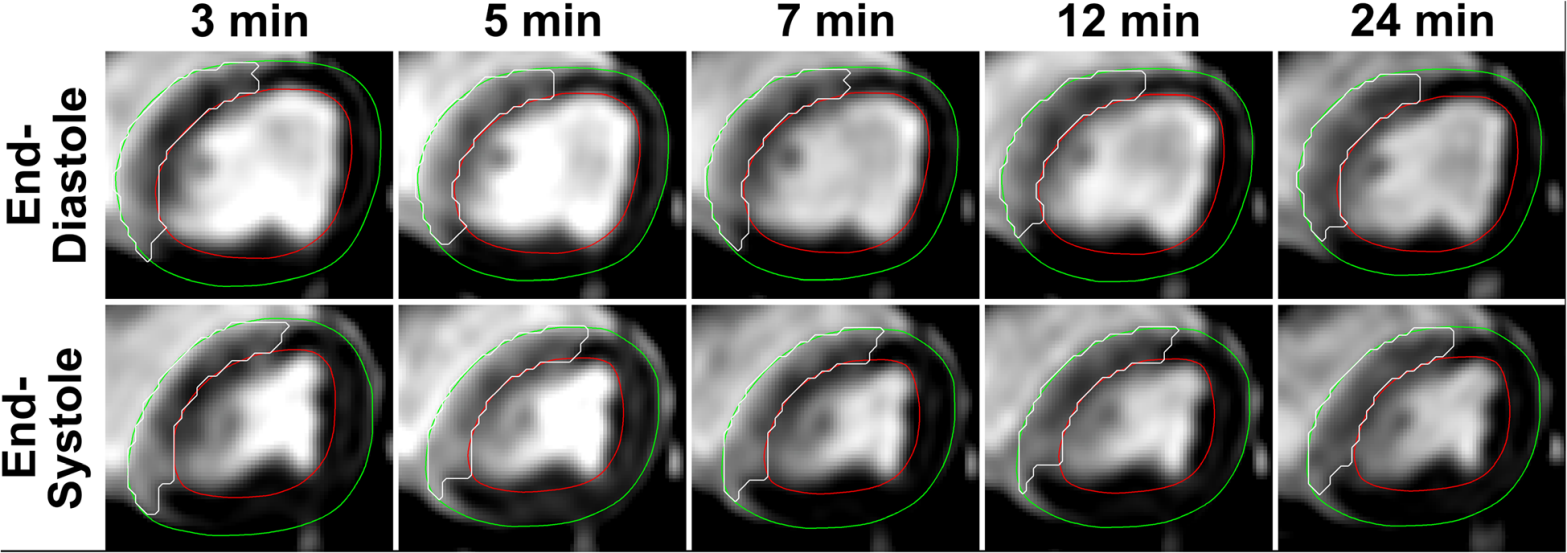
Myocardium at risk (MaR) by CE-SSFP at various time points after contrast agent administration. The upper row shows CE-SSFP at the end-diastolic (ED) time-frame and the bottom row at the end-systolic (ES) time-frame. The green line denotes epicardium, the red line endocardium and the white line MaR. Note that there is no change in the extent of MaR over time. Note also that there is a hypointense core within MaR at 3 min not visible at later timepoints. This could be due to slower contrast filling to this area, possibly because of microvascular obstruction

**Fig. 5:**
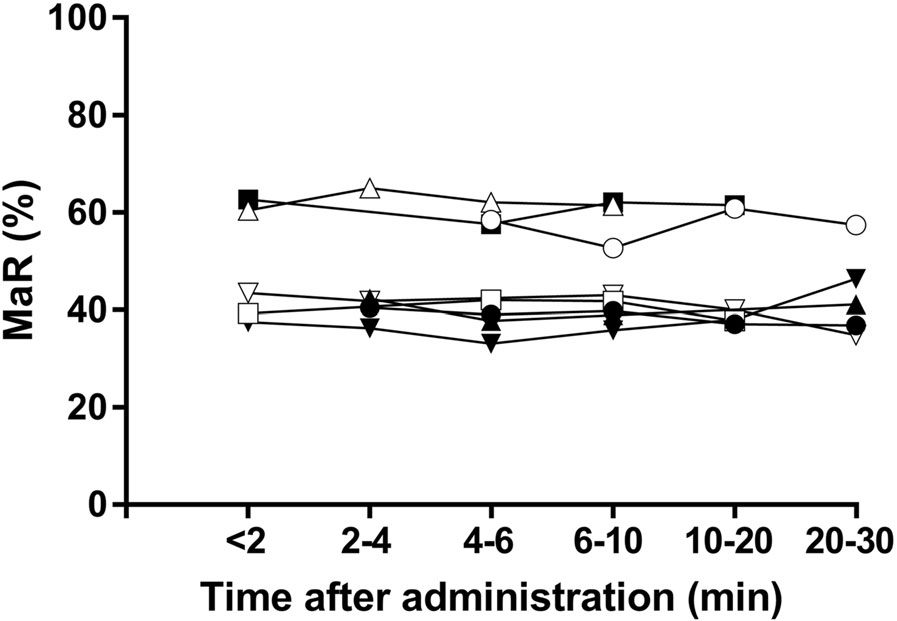
Myocardium at risk (MaR) by CE-SSFP over time after contrast agent administration in single midventricular slices. There was no change in MaR up to 30 min after contrast agent administration (*p* = 0.95)

Images of MaR over time in one animal are seen in Fig. 4. On average, the MaR at baseline did not change over 30 min after contrast injection (*p* = 0.95). Bias for different times after contrast injection compared to baseline is shown in Fig. 5. Inter-observer analysis for MaR over time showed a low bias (0.9 ± 5.7%, *r* ^2^ = 0.77).

## Discussion

This study validates CE-SSFP in an experimental setting and shows that this technique can be used to measure MaR with high accuracy and precision in vivo and ex vivo. It also shows that CE-SSFP for measurement of MaR is stable at least up to 30 min after contrast injection.

### Potential mechanisms for detecting MaR with CE-SSFP

The increased signal found in the entire MaR is likely explained by a shift in the T2/T1 ratio that determines signal contrast for the balanced SSFP sequence [19]. Without the contrast agent the tissue edema results in a higher ratio as T2 seems to be proportionally more increased than T1 [20]. At certain concentrations of contrast agent myocardial T1 may decrease proportionally more than T2, thus further increasing the T2/T1 ratio. It has been shown that the MaR has an increased distribution volume of contrast agent compared to remote myocardium [21–23], which could allow for the contrast agent to affect the T2/T1 ratio to a greater degree in MaR yielding an observable difference in signal intensity. Further studies will be needed to elucidate the mechanisms behind the tissue contrast between MaR and remote myocardium in CE-SSFP.

The single-slice data over time shows that CE-SSFP MaR imaging can be performed from less than 2 min up to 30 min after contrast agent injection with no detectable changes in the extent of the MaR. This suggests a rapidly established equilibrium of contrast agent between the extracellular compartments (in blood, remote myocardium, MaR, infarct) in which the contrast agent is distributed (excepting areas of microvascular obstruction wherein contrast wash in is delayed or wholly obstructed). Previous studies have shown an increase in T1-relaxation times that is proportional between the extracellular compartments over 30 min after contrast injection which corroborates this finding [21, 22, 24, 25].

### Previous studies using CE-SSFP for MaR

Sörensson et al. validated CE-SSFP using MPS as reference standard in patients with ST-elevation myocardial infarction [9]. They found a bias of 0.5 ± 5.1% with a correlation of *r* ^2^ = 0.78. In addition, Ubachs et al. showed good agreement between T2-STIR and CE-SSFP for assessment of MaR in a single-center, single-vendor setting [10]. More recently, CE-SSFP has been shown to be more robust than T2-STIR in a multi-center, multi-vendor setting [6]. Furthermore, in accordance with the findings in the present study that MaR is constant over time early after contrast injection, Ubachs et al. showed a constant relationship between MaR by pre-contrast T2-STIR and CE-SSFP acquired 2–12 min after contrast injection [10].

Thus, CE-SSFP offers a robust method for determination of MaR which has made it the method of choice for several clinical cardioprotection trials [11, 12, 26].

### Experimental and clinical implications

MaR has been determined experimentally for several decades but has required separate modalities such as histopathology dyes, microspheres, and radioisotope imaging, making image co-registration a challenge [4, 21, 22, 27, 28]. Using CE-SSFP to determine MaR abolishes the need for separate modalities as both infarct size and MaR can be assessed in one single CMR session. Thus, co-registration of infarct and MaR can be performed accurately on a slice-by-slice basis. Furthermore, CE-SSFP cine imaging enables assessment of both MaR and myocardial function which enables calculation of myocardial salvage and myocardial salvage index and reducing the number of subjects with preserved power [29]. Since myocardial function is an essential part of most in-vivo protocols, the use of CE-SSFP shortens the CMR examination as it abolishes the need for separate scanning for assessment of MaR and myocardial function. In addition. The ability to use ex-vivo or in-vivo CE-SSFP interchangeably confers the option to perform only ex-vivo scanning and forego the more complex in-vivo scanning when determining myocardial salvage in experimental studies.

#### Limitations

The findings in the present study should be interpreted in the light of the following limitations. First, when comparing MaR-size over time, single midventricular slices, and not full LV coverage, were acquired. Thus, the total MaR was not assessed repeatedly, rather the percentage of MaR in a single slice. Second, the data over time after contrast-injection could not be acquired at all timepoints for all animals. This was due to both technical (e.g. triggering problems) and medical reasons (e.g. hemodynamic instability requiring intervention), interrupting imaging acquisition in some animals. Third, manual delineation was used to assess MaR. Manual delineation has, however, been validated against MPS [9]. In future studies, techniques such as T1- or T2-mapping sequences could potentially allow automatic or semi-automatic evaluation of MaR [20, 30], however, more standardization and validation is needed before these techniques can be used in clinical trials.

## Conclusion

Contrast-enhanced SSFP cine CMR imaging can be used to measure MaR, both in vivo and ex vivo, in a porcine model with good accuracy and precision over the first 30 min after contrast injection. This offers the option to use the less complex ex-vivo imaging when determining myocardial salvage in experimental studies.

## Abbreviations

AMI: Acute myocardial infarction
CE-SSFP: Contrast-enhanced steady state free precession
CMR: Cardiovascular magnetic resonance
LGE: Late gadolinium enhancement
LV: Left ventricle
LVM: Left ventricular mass
MaR: Myocardium at risk
MPS: Myocardial perfusion single photon emission tomography
T2w: T2-weighted
TE: Echo time
TI: Inversion time
TR: Repetition time
